# Reviewed article: Araújo LBS, Sousa RA, Aguiar BGA, Mendonça VJ, Araújo OD, Tajra FS. Spatial and temporal distribution of neglected tropical diseases: overlap analysis, Piauí, 2013-2022. Epidemiol Serv Saude. 2026:35;e20250373

**DOI:** 10.1590/S2237-96222026v35e20250373.a

**Published:** 2026-03-16

**Authors:** Anderson Fuentes Ferreira

**Affiliations:** 1 Universidade Federal do Ceará, Fortaleza, CE, Brazil

## Peer review A

## Questionnaire

Does the manuscript contain new and significant information to justify publication? Yes

Does the Abstract (Summary) clearly and accurately describe the content of the article? No

Is the problem significant and concisely stated? No

Are the methods described comprehensively? No

Are the interpretations and conclusions justified by the results? No

Is adequate reference made to other work in the field? Yes

## Manuscript Structure

Length of article is: Adequate

Number of tables is: Adequate

Number of figures is: Adequate

Please state any conflict(s) of interest that you have in relation to the review of this paper. None

Interest: Average

Quality: Average

Originality: Average

Overall: Average

Recommendation: Major Revision

## Comments

Primeiramente gostaria de agradecer a oportunidade de dar parecer para o artigo “Distribuição espacial e temporal das Doenças Tropicais Negligenciadas no Piauí, 2013 a 2022: análise de sobreposição”. As Doenças Tropicais Negligenciadas são um problema de saúde publica relevante para o Brasil, especialmente em regiões de alta endemicidade, como é o caso do estado do Piauí.

Dito isso, vou fazer algumas considerações com intuito de melhorar a qualidade do artigo.

Título

Acredito que o titulo precise ser reformulado, para refletir o objetivo do artigo.

Resumo

No método, deixar claro o tipo de estudo.

Introdução

Sugiro aos autores incluíres o referencial para inclusão da tuberculose como uma Doença Tropical Negligenciada.

Falta um paragrafo de introdução sobre a definição do que são estas doenças, e apresentação básica da epidemiológica das Doenças Tropicais Negligenciadas no Brasil, para contextualizar o cenário do estado do Piauí.

Na página 5, ao final da introdução, os autores colocam as perguntas a serem respondidas. Isso não é usual, visto que já existe um objetivo para nortear o método, resultados e discussão.

Método

No desenho de estudo, deixar claro o tipo do estudo (Ecológico, transversal ou outros).

Na fonte de dados, não é necessário incluir os CID10 das doenças analisadas, visto que estas são de notificação compulsória. O CID10 precisariam ser informados para um estudo com dados de mortalidade, com busca na base do Sistema de Informações sobre Mortalidade (SIM).

Na analise dos dados, sugiro aos autores calcularem as taxas de incidência e prevalência ajustando pela idade, considerando a população brasileira como padrão. Com isso, outros estudos poderão comparar os resultados deste estudo.

Resultados

Para a descrição geral, é recomendado apresentar o número seguido do percentual.

Na tabela 1, de forma agrupada, os autores poderiam calcular as taxas de incidência e prevalência, para avaliar a magnitude destas variáveis nos eventos estudados.

Cuidado com as padronizações, em alguns pontos os autores usam “habitantes”, em outros “hab.”.

De forma geral, os resultados ficaram bem longos. Sugiro aos autores apresentaram os principais destaques. Os resultados apresentados nortearam a discussão.

Discussão

No segundo paragrafo da discussão, os autores comparam o sexo e a faixa etária. Baseado apenas em percentuais não é possível esta discussão. Sugiro aos autores aprofundarem as discussões, em relação aos achados sociodemográficos, assim como para as incidências com as doenças.

De forma geral, a discussão precisa ser melhor fundamentada, com base em outros estudos sobre Doenças Tropicais Negligenciadas, em especial para hanseníase.

Ao final da discussão, formar as limitações do estudo.

Tabelas e figura

Na tabela 1, calcular a incidência e prevalência global segundo as variáveis.

Na figura 2, sugiro padronizar o padrão de cores usados nas categorias dos mapas.

Na tabela 2, sugiro aos autores calcularem a variação percentual anual para as variáveis inclusas na tabela 1, de forma global.

